# Experiences of formal caregivers of elderly inpatients with physical disabilities in China: a qualitative study

**DOI:** 10.1186/s12912-024-02019-3

**Published:** 2024-06-07

**Authors:** Li-li Sun, Li Zheng, Liu-liu Chen, Zhao-di Wang, Qian Li, Li Liu

**Affiliations:** 1 Department of Nursing, The First Affiliated Hospital, Zhejiang University School of Medicine, Hangzhou, 310000 China; 2 School of Health, Zhuhai College of Science and Technology, Zhuhai, 519000 China

**Keywords:** Carer, Formal caregiver, Caregiver burden, Qualitative research, Aged, Disability, Inpatients

## Abstract

**Aim:**

To explore the views and experiences of formal caregivers caring for older inpatients with physical disabilities.

**Methods:**

It was a qualitative phenomenological study. Using purposive sampling, twelve formal caregivers were chosen in a tertiary comprehensive hospital in Hangzhou, China. Semi-structured, face-to-face interviews were conducted, guided by open-ended questions that focused on gaining rich insights into participants’ views and experiences. Coding reliability thematic analysis was used to guide data analysis and categorize, based on Lazarus and Folkman’s theory of transactional coping.

**Results:**

Four themes emerged from the data analysis: (1) Caregiving Threats. (2) motivations. (3) Responsibility Management. (4) Fear.

**Conclusion:**

Despite facing significant pressure at work, formal caregivers of elderly inpatients with physical disabilities possess the drive and various coping strategies to excel in their role. Identifying caregivers’ experiences of care can be helpful in improving resilience to stress and maintaining stability in formal caregivers.

## Background

The number of adults aged 60 is expected to increase to 1.4 billion by 2030 and 2.1 billion by 2050 [1]. Disability and chronic disease are closely linked to old age. 19.3% of adults aged 65 and over reported having a lot of difficulty or being unable to do anything at all in at least one area of functioning [2]. This results in a full-time caregiver staying with the disabled older person. While, with the decrease in family size, young offspring are struggling to balance caring responsibilities and employment, leading to the hiring a formal caregiver to take over care tasks originally performed by themselves.

Formal caregivers, playing an important support role in the aged care system [3], who function similarly to healthcare assistants but are unlicensed workers, are called medical nursing assistants in Mainland China [4]. Formal caregivers are also known as paid caregivers, home health aides, and personal care attendants, and they are hired by patients or the patients’ family to work in various long-term care settings, such as residential homes, continuing care hospitals, and private homes [5, 6]. From 2019 to 2029, this occupational group will add an estimated 1.3 million formal caregivers to meet rising demand, which is more than in any other occupation [7]. However, the roles of formal caregivers vary by location and context, within legal, regulatory, and institutional boundaries [8]. In hospital wards, formal caregivers are actively needed by different stakeholders, especially the elderly with self-care deficits [9], the elderly’s family members, and the medical staff. They are regarded as the first line of contact for patients in lieu of their families, constantly looking after the elderly, being both mindful and responsive to any needs and helping out with the patient’s activities of daily living, which involve bathing, dressing, transferring, eating, and toileting. Accordingly, formal caregivers are pivotal members of the multidisciplinary team, impacting both the elderly’s quality of life and the medical personnel’s evaluation of a patient’s health and recuperation [10–12]. . They are also a necessary addition to the nursing workforce, helping to address the shortage of personnel [13]. Moreover, formal caregivers consider patients as clients, interacting with the family frequently as trustworthy persons, especially in the case of clients suffering from Alzheimer’s disease [14, 15].

However, providing 24/7 close supervision of patients with disabilities is a demanding task [8, 16]. Formal caregivers, who are considered “Invisible Patients” [17], suffer from a high physical and psychological burden [18, 19]. The uncontrollable and disruptive behavior of the elderly with disabilities, which contributed to the formal caregivers’ multifaceted burdens in stressful situations, affected their health and quality of life [16, 20, 21]. Gao et al. [22] systematically reviewed 35 studies with a total of 3,268 caregivers and found that caregivers typically received 2.4 to 3.5 fewer hours of sleep per week than the non-caregiving controls. In addition, formal caregivers were found to be under immense psychological pressure [23]. Traditional discrimination against formal caregivers leads to professional shame, and low professional identity brings subjective psychological pressure [24]. A cross-sectional study on the health-promoting lifestyles of 634 formal caregivers from eastern, central, and western China found that it was poor for 14.04% of them and average for 45.11% [25]. In this study’s review of the literature, previous studies mainly focused on the quantitative analyses of formal caregivers’ burden and its impact, but they did not address how they coped with their burden and how they felt during their provision of care.

The experiences are seen as a transaction between an individual and their environment. Lazarus and Folkman’s Transactional Model of Stress and Coping [26–28] suggests that understanding how individuals perceive and analyze stress is critical in their response to stress-inducing factors. Positive outcomes are achieved with a successful response, otherwise failure to respond effectively leads to negative outcomes. Effective improvement of positive response can help caregivers adapt to their role of care [29]. Karen A Roberto, et al. [30]. applied Lazarus and Folkman’s Transactional Model of Stress and Coping to revealed 10 types of behavioral expressions of dementia to engaging in necessary activities of daily living, and caregivers’ adaptive strategies for managing behaviors.

Therefore, this study explored the formal caregivers’ experiences of caring for elderly inpatients with physical disabilities in a Chinese tertiary hospital. A qualitative methodology was employed to uncover and grasp formal caregivers’ experiences and their significance based on the theory of Lazarus and Folkman’s Transactional Model of Stress and Coping. The findings of which could be implemented into interventions in the future to support this group of people. Furthermore, the discoveries may have far-reaching implications for providing guidance on how to offer a focused, prompt assistance for formal caregivers to lighten the load and enhance the quality of care.

## Methods

### Study design

Phenomenology is a well-established qualitative research approach that is widely used to explore the lived experience of an individual in relation to a particular phenomenon [31]. Therefore, a qualitative approach was adopted based on semi-structured, in-depth, face-to-face interviews with the formal caregivers of dependent elderly inpatients to understand their lived experiences. The Consolidated Criteria for Reporting Qualitative Research (COREQ) checklist (See Supporting Information File S1) was used to meet the standards of high-quality research [32].

### Participants

Eligible participants for this study were formal caregivers who were living with and caring for the elderly with physical disabilities in a tertiary hospital in Hangzhou, Zhejiang Province, China. The purposive sampling method, combined with a maximum variation [33] in the sample (e.g., sex, age, education level, beliefs, length of care, etc.), was used in the selection of participants between May 1 and July 30, 2022. The inclusion criteria for this study were as follows: (a) primary caregiver of physically handicapped, hospitalized elderly patients ≥ 65 years old; (b) paid for by families or patients; and (c) could understand Mandarin and speak it fluently. The participants continued to be enrolled until new interviews provided no additional information, which is considered a sign of data saturation, the gold standard for sample size [34].

### Data collection

A semi-structured interview with open-ended questions was developed by first author (LLS) based on a literature review and revised on the basis of group discussion. Then, a pilot test was conducted with three interviewees who were not included in the final study. Finally, some minor revisions were made by two researchers (LZ, LLC), who were experienced in qualitative interview techniques. Table 1 provides an outline of the semi-structured interview guide. To gain the most insight and clarity, probing questions were asked [35], such as “What happened next?”, “What were you thinking then?”, “How did that affect you?”, “Can you please give an example?”, and “What do you mean by that?”. The data collectors (LLS and QL) were primary researchers who were also health professionals with training in qualitative interview techniques. Qualitative data collection methods included face- to- face individual interviews. The interviews were audio-recorded using telephone recording software, and conducted in a quiet, meeting room in the hospital, only the participant and the interviewer were present during the interviews. The participants were given a written informed consent form to sign at the beginning of the interviews. They were told that the interviews would be recorded, their anonymity would be safeguarded, and that they could quit at any time without any retribution. Prior to the interviews, the participants also completed a short demographic survey. After the interviews, the participants were offered a small hand hygiene product to thank them for their time. Interviewees were asked to record field notes [27, 36], which contained details such as mood, actions, expressions and meaningful glances not mentioned in the interviews. Moreover, Interviewees were asked to immediately transcribed verbatim and manually cross-checked for accuracy within 24 h by two researchers. Twelve formal caregivers (P1 through P12) were interviewed. The interviews lasted between 43 and 77 min. All the interviews and interview diaries were saved as digital files on a secure network computer.

**Table 1:** Outline of the semi-structured interview

Q1.	What are your responsibilities on a daily basis?
Q2.	What is your opinion of your job? Can you talk about your feelings?
Q3.	What is the opinion of your work held by your relatives and friends? Can you provide some examples?
Q4.	What kinds of experiences do you find challenging in caring for the elderly with a disability? Can you provide some detailed examples?
Q5.	How do you deal with these challenges? Can you provide some detailed examples?
Q6.	What kinds of things make you sad in caring for the elderly with a disability? Can you provide some detailed examples?
Q7.	How do you deal with these sad feelings? Can you provide some detailed examples?
Q8.	How do you feel about elderly inpatients with physical disabilities? Can you talk about your feelings?
Q9.	How do you feel about your own family? Can you talk about your feelings?
Q10.	How have you changed since doing this job? (physical change or psychological change)
Q11.	If you had the chance to choose this job again, would you continue to do the job of caring for the elderly with disabilities? Why or why not?
Q12.	Is there anything else that you would like to add?

### Data analysis

Numerical codes (P1 through P12) were assigned to the participants, and any personal information was removed from the transcripts of the interviews to guarantee anonymity. Investigator triangulation [37], where more than two researchers were involved in the analysis process to support the validity of the findings and to meet expectations. Coding reliability thematic analysis, thoroughly described by Braun and Clarke [36, 38], was then used to recognize, appraise, and summarize themes within qualitative data [39]. This approach is oriented to providing reliable and accurate information from the data, aligning with a realist epistemological perspective. Data familiarization was applied to start the analysis process, LLS and LL scrutinized transcripts and diaries of interviews several times to achieve a broad understanding and minimize prejudices independently [35]. Then, they individually analyzed the data, manually coding each line, relying solely on the words of the participants to avoid introducing any preconceptions [40]. They met regularly to ensure inter-rater reliability. To find consensus amongst researchers, they also discussed the development of codes and themes derived from the data. A third researcher (LZ) was there when the codes disagreed. A finalized codebook was created containing a theme label, definition, as well as detailed examples and descriptions of what is included and excluded within each theme.

**Fig. 1:**
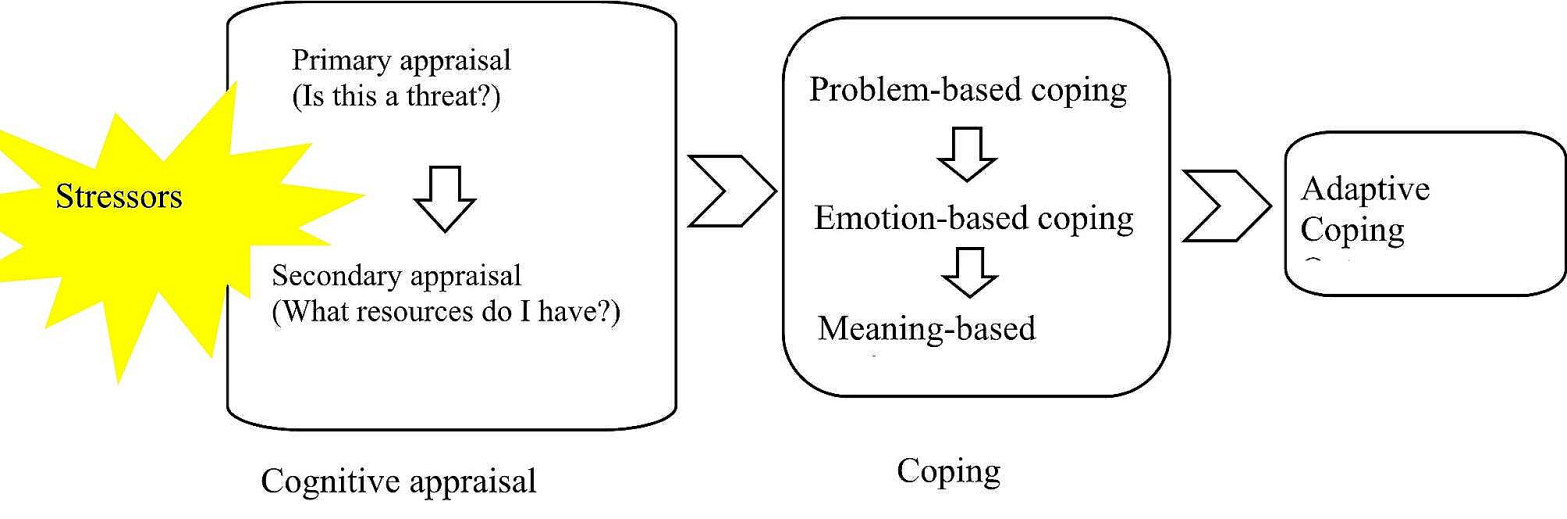
Modification of the Transactional Model of Stress and Coping. The combination of stressors stimulates the caregiver’s cognitive appraisal of the job, which consists of primary appraisal (e.g. is this a threat? ) and secondary appraisal (e.g. what resources do I have? ). These appraisals lead caregivers to develop appropriate coping strategies, including problem-based, emotion-based and meaning-based coping. Finally, the individual will make an adaptive change

In a subsequent analytic step, Modification of Lazarus and Folkman’s Transactional Model of Stress and Coping was used to guide the organization of themes and sub-themes [41]. According to this theoretical framework, two processes, including cognitive appraisal and coping, have been identified as key mediators of stressful human-environment relationships and their immediate and longer-term consequences. Cognitive appraisal, commonly known as primary or secondary appraisal, is a process of assessing whether the environmental experience is relevant to health and, if so, how it affects it [26, 42]. The process of coping is defined in terms of the continuous change of cognitive and behavioral efforts in order for the understanding of the situation to deal with the specific external and internal demands. Approaches to coping typically fall into three major areas: problem-solving, emotional regulation, and deciphering meaning [26, 27]. Then after the two processes, there is also a change in the adaptation of the individual, as shown in Fig. 1.

## Results

### Participant characteristics

Twelve formal caregivers participated in the study, three men and nine women. The mean age of the participants was 59.42 ± 4.44 years (range 53–65 years). They were all from rural areas, far from Hangzhou, the city where they did their work. Five participants had primary education, four had secondary education, one had tertiary education, and two had no education. The length of time spent caring for elderly patients ranged from a minimum of one year to a maximum of 24 years. All had the experience of looking after highly dependent elderly individuals in a hospital, and four of them had looked after more than 10 elderly inpatients. Ten participants were supervised by an official institution, SanTi, an external company located in the hospital but independent of the hospital, while two participants were employed by their patients’ families based on the recommendations of a housekeeper. Two participants adhered to Buddhist and Christian faiths, respectively, while the rest practiced no religion. The demographic characteristics of the eligible participants are shown in Table 2.

**Table 2:** Demographic characteristics of the eligible participants

Characteristic	Categories	Frequency (*N* = 12)	Characteristic	Categories	Frequency (*N* = 12)
Gender	Male	3	Religion	None	10
	Female	9		Christian	1
Age	50–54	2		Buddhist	1
	55–60	4	Institutions	SanTi^*^	10
	60–64	5		Housekeeping company	2
	65–70	1	Length of Service (in years)	1–4	6
Residential Area	Rural	12		5–9	1
Education Level	None	2		10–14	1
	Elementary school	5		15–19	1
	Middle school	4		≥ 20	3
	High school	1	Number of Disabled Elderly Inpatients Cared For	1–3	7
Marital Status	Married	11		4–5	1
	Widow	1		>10	4

*Note* *The name of an external company responsible for the management, deployment and training of formal caregivers, located within the hospital but independent of the hospital

### Themes

After data analysis, four themes were identified: Caregiving Threats, Motivations, Responsibility Management, and Fear. Seven sub-themes also emerged: Physical Harm, Psychological Pressure, Self-efficacy, Family Support System, Work-Related Value, Problem-Solving, Psychological Acceptability. A description of each theme, the participants’ codes and quotes were shown in Table 3.

**Table 3:** Four major themes that emerged from codes and quotes

Themes	Codes	Quotes
Caregiving Threats	Sleep Deprivation	“It’s hard, I can’t sleep at night…my eyes are dry. He needs daily infusions, and last until midnight. After that, he’s usually ready to get out of bed and go out. But if he were to get up, he wouldn’t even be able to walk…. I can only sleep a little at night, sometimes a couple of hours, sometimes less than an hour.” (P10)
	Radiation exposure	“Every time a patient has a CT scan, you know about radiation, a doctor said one of members should stay in the room, you know…only I was there….” (P12)
	Pain	“He stood up, and I knew I’d support him, but he resisted and hit me with a crutch. Even though he was fighting me, I couldn’t lose my grip, I was afraid he would fall.” (P7) “ He (the old man) is very heavy, I roll him over with one hand and pat his back with the other, and my hands hurt. My hands hurt now…” (P9) “Although I’m strong…. But I felt exhausted, I hurt my waist. Now if I use a bit of strength, it would be painful in my back.” (P10)
	Guilty conscience	“You can only take care of one. If you take care of this, you can’t take care of the other.”(P10) “My mother is also hemiplegic, and she stays at my sister’s. Sometimes when my mother calls me, I’m in a bad mood and I cry and I wonder why I can’t take care of my parents.” (P5)
	Inferiority	“I talked to my family every day. If they asked me [what I was doing], my answer was, a cleaner. If they knew, they’d feel sad. I’d take it for myself. I wouldn’t tell them.” (P9) “Doing this job, I have low self-esteem. When my friends and relatives asked me about it, I told them that I were doing hygiene and I did not dare to say that I were serving people. If they knew that I served the elderly, they would laugh at me.”(P5)
	Isolation	“I have always been depressed. I’ve never laughed, now I just look at my phone and I have a few friends, but I don’t talk to anyone…they [my friends] were chatting in the WeChat group, but I didn’t participate. I don’t like to talk or laugh with other people….” (P5)
	Fear	“I have to do well once I’ve taken care of my patient, no mistakes, no losses, I’m afraid a little mistake will happen. I can’t do anything wrong or harm, can I? No” (P5) “You know, hospitals are always full of germs, a bit of a worry, a bit of a thought, like I’m trying to get lucky…. He [the elderly patient] had bacteria in his urine, and I had to wash my hands every day after helping him, and before I ate, I washed my hands. It was a bacterial infection, and I was a bit scared.” (P7)
Motivations	Conscientious	“The patient is innocent, and I don’t care about his family members. I do it according to my conscience. Everyone else [other caregivers] must feel the same way….” (P5) “This work was hard, but it was ‘okay’ if you were used to it…. It’s about setting a good example for my granddaughter, she was proud of me….” (P2)
	Caring Experience	“I had been a barefoot doctor in my town when I was young…. Whatever comes my way, I’m ready to tackle it.” (P2) “I had been a barefoot doctor in my town when I was young…. Whatever comes my way, I’m ready to tackle it.” (P2)
	Healthy body	“I am fit and have no difficulty in taking care of the elderly… As long as I’m physically able to do so, I’ll continue to do so.” (P4) “I’m healthy, and I’m doing well…. I manage to go on….” (P9)
	Offspring’s Online Companion	“My daughter and son knew it was a 24-hour job, and it was tiring, so in winter they sent me some local dishes, and during Chinese New Year they ordered food for me over the phone.” (P7)
	Parents’ support	“When I couldn’t go back home, my father said, ‘Okay….’ He said, ‘I understand you, and you don’t have to come back’.” (P3)
	Siblings’ Help	“…my dad had a car accident, suddenly he couldn’t walk; my sister took care of him for a few days; my brother came back for a few days; my sister often video-chatted with me….” (P10)
	Couples’ Support	“My wife looks after elderly people in another ward. She admires my work, I’m fine, I told her, the nurses, the doctors, no one said anything bad about me, they all complimented me….” (P12)
	Pay satisfaction	“To make it, even though I was embarrassed at times, I knew that I had to keep going and find ways to make money.” (P11)
	Being approved	“When the doctors do their rounds and I have to give [the patient’s condition] so that they know what the patient’s specific condition is…. Both the doctors and leaders trust me.” (P2) “When I stood at the door, she [the elderly patient] couldn’t see me, and she would call me, ‘Zhang-Zhang…’, repeatedly until I answered. If she was reading a book, she would always read it, then look at me, and then read on. She was afraid I’d leave.” (P4) “The daughter is worried about me going home. When I went back to the house for eight days, she called me four times….” (P3).
	Emotion benefits	““My sick mother died in hospital. Sometimes I look at her (the patient) and I feel like I’m back with my mother”.” (P1) “I was in contact with him [the elderly patient] for a long time, compared to my parents…. It was a 24-hour shelter, with some emotion.” (P2)
Responsibility Management	Regularity exploration	“I know everything about him, eating, temperament, bowel movements, all regular. I’ll get it in time… I’m basically okay with whatever it is.”(P7) “It’s difficult if you don’t explore (the law)… No matter how much I sleep, when he goes ‘hum, hum’, I know he’s being re-positioned” (P1)
	Skills exploration	“ I looked after an elderly woman who weighed over 200 pounds… I also use dexterity; it is impossible to move a patient by brute force alone. All these methods are done by myself, by slowly finding them out.” (P10). “When we meet, you talk about your patient, I talk about mine, how can we take good care of him, as we have been taking care of him for a long time, we understand in general, but some people who have not taken care of the elderly, they do not know, so we will talk to them. We communicate together to help each other.”(P12)
	Rationalization	“We came from a rural area, we took care of the men, wiped their bodies, we felt embarrassed…. It’s a shy thing, and it’s more embarrassing…but he’s a patient, male or female patient, you have to take good care of them.” (P10) “He [the elderly patient] was like a child, really, if you got angry with him, you would make things worse. You just have to persuade him, sometimes you have to praise him, just like a two- or three-year-old child.” (P8) “He’s noisy when he’s wet on the skin, and when I’m wet on the skin I feel uncomfortable too. So you change them—the sheets and the urine pads—in time, right?” (P2)
	Religion	“It is good to help sick people. He [the elderly patient] was given to me by God…. I’m doing it all the time.” (P3)
Fear	Fear of becoming a disabled elderly person	“I would be afraid if I got old. I’ve seen old people dying [in hospitals], The old man [the elderly patient], is pitiful and lonely. When the old people died, there were no families around.” (P4) “I have no money, When I was old and sick, I would go to the mountain and drink medicine by myself, and I will not tell anyone about it.” (P5)
	Fear of no followers	“When we were old like them [the elderly patients], nobody did it for us. In the one-child generation, a couple is responsible for four old people, and if they were all in bed, the couple wouldn’t have time for them.” (P4)

### Theme 1: Caregiving Threats

Based on the Transactional Model of Stress and Coping, individuals perceive the seriousness or threat of specific stressful transactions, and then identify risks through primary appraisals. Most participants described caring for those elderly who lacked the capability to do daily activities as very exhausting. Two sub-themes supported the theme of Caregiving Threats: (1) Physical Harm; and (2) Psychological Pressure.

### 1–1 Physical harm

#### Sleep deprivation

Almost all the participants reported difficulties in getting a full night’s sleep, because they had to provide nightly care and respond to the needs of their clients. Therefore, many participants experienced sleep deprivation because of their clients’ insomnia [43], especially if their clients’ condition is deteriorating, or they are delirious, depressed, or affected by medical conditions. Moreover, formal caregivers also need to be awake during the day to monitor IVs, clean skin, and attend to other needs of the elderly.

#### Pain

Almost all the participants described their experiences of heavy daily work, including transporting and repositioning patients. Repositioning a patient regularly is a highly effective approach for preventing pressure injuries, especially for those who are totally dependent on assistance with activities of daily living. In addition to repositioning, transferring a patient is a common manual handling task that involves moving a patient from a bed to a chair, wheelchair, toilet, etc. Consequently, physical pain (e.g. in the back or wrist) is a familiar difficulty for caregivers. To make themselves more comfortable, some participants wore waistbands or musk pain patches. In addition, they may also encounter workplace violence [44] from clients suffering from severe behavioral disorders due to Alzheimer’s disease or other neurological disorders.

#### Radiation exposure

A number of participants reported concerns about the risk of radiation exposure when accompanying their clients for CT scans or any other radiation-emitting test. If the client’s condition was critical or changing, the doctor would refer the patient for further tests. They then needed to be in the examining room to make sure their client was safe.

### 1–2 Psychological pressure

#### Fear

Most of the participants expressed that they had to take responsibility for the safety of their patients. Their routine of caring for elderly patients in the hospital was carried out repetitively, carefully, and with fear. Especially when the elderly patients were in critical or deteriorating condition, formal carers experienced much more fear and depression. In addition to this, the very environment of the hospital also made them feel fearful. Some of the participants expressed that they might become infected.

#### Inferiority

Some patients or family members thought they were paying for services, so they believed they should have a sense of superiority, just like a buyer (in contracts). For formal caregivers, however, providing personal service like a servant is not a respectable job. It is inferior and looked down upon. *“People look down on us for doing this.” (P4)* If the employers didn’t respect or trust them, formal caregivers would feel even more inferior. To combat the feelings of inferiority that come with the job, several participants avoided talking to their families and relatives about what they did, instead saying they were cleaners.

#### Isolation

The formal caregivers who had left their families were asked to spend day and night with their clients in another town, looking after them, talking to them, sleeping next to them, and eating with them. But for the caregivers, they were still very lonely inside. *“It must be very lonely to have nothing to say to them (their clients).”*(P12) Particularly during the spread of COVID-19, the residential unit was instructed to isolate itself from management, and they were even instructed to avoid visits, leading to social isolation. *“Been round the ward all day, not talking to anyone… ”*(P9) Prolonged isolation also causes caregivers to feel inferior, lack confidence, become depressed and fear socializing.

#### Guilty conscience

Hospital care for the elderly took the formal caregivers away from their homes and families. The participants were guilty that they had to care wholeheartedly for other people’s parents instead of their own, especially when their own parents required assistance. Particularly around the holidays, such as Christmas in mid-autumn and Chinese New Year, the loneliness of being away from their families weighed heavily on them, deepening their guilty conscience. *“It’s all under one roof, 24 hours a day; I’ve been looking after this old man for years, longer than my parents have been together.”*(P1) Many participants expressed helplessness about the psychological burden of guilt. Crying alone was the only way reported by the participants to release this guilty conscience.

### Theme 2: Motivations

Every coin has two sides. It is an undisputed fact that caring for an elderly person with a disability involves a great deal of physical and psychological strain. But formal caregivers are also highly motivated to carry this burden and to continue the job. The motivation can help a person to achieve a goal [45]. The interviews revealed several motivations including intrinsic and extrinsic motivation among the participants. Three sub-themes supported the theme of Motivations: (1) Self-Efficacy; (2) Family Support System; and (3) Work-Related Value.

### 2 − 1 Self-efficacy

#### Conscientious

For non-family caregivers, conscience is the most important personal quality that determines the quality of life of the elderly. Some of the participants described how they were compassionate towards the elderly and showed great willpower when faced with a heavy burden. Almost all participants talked about being conscientious in order to be competent. They believed that if they put their heart and soul into helping the elderly, they would not feel sorry for the elderly who were suffering, and they would be worthy of their income, thus earning the respect of others. What’s more, some participants were proud of caring for the sick, which was believed as a valued job, and hoped their offspring would be able to care for the elderly in the future.

#### Healthy body

A healthy body is a prerequisite for doing anything. Maintaining a healthy body was one of key factors allowing an individual to believe that he or she could fully accomplish various obstacle tasks. All participants described themselves as being free of illness and in good health and believed that a healthy body was the main reason why they could continue to do this work. Therefore, some participants expressed their determination to continue as long as they had a healthy body, even though it was a job full of dangers and challenges.

#### Caring experience

The formal caregivers’ previous experiences of caring for people outside the hospital gave them a high level of confidence in their competence. Two female participants articulated their rich experiences of caring for their parents. In addition, one male participant who was a barefoot doctor provided health education and preventive care to his rural neighbors in a culturally appropriate manner. On the basis of a wealth of experience in caring, some participants claimed that they had the competence to take on their role.

### 2–2 Family support system

#### Parents’ support

Almost all the participants were from rural areas, far from Zhejiang Province, so they had no chance of staying with their parents or taking care of them. Gaining parental understanding and support were a source of comfort, allowing them to focus on their duties.

#### Siblings’ help

The participants, especially those with living parents, worked and rarely returned, while their siblings played an important role in the family. Although they gained their parents’ understanding, they also felt guilty of not being able to be there for them, especially when their parents were sick or in need. But because of the help of their siblings, they could be relief, and work with more peace. Not only did their siblings take care of their parents on a daily basis, but they also provided timely and effective help in unexpected situations.

#### Offspring’s online companion

Thanks to networking, communication between people is convenient and effective. Almost all the participants said that they used the WeChat social media [46] to chat with their children every day via voice and video. Some of the participants’ offspring also sent love to their parents via the network.

#### Couples’ support

Two of the participants had the same job as their spouses. Both of them and their spouses were in different hospitals. These couples’ similar experiences not only helped them to understand and encourage each other, but also to be positive in their work.

### 2–3 Work-related value

#### Pay satisfaction

Almost all the participants said that the motivation that led them to do the heavy work was that they received “pay satisfaction” for their work. In the past, because of age or education restrictions they worked in the fields or in the city at a temporary job, which made them tired but less paid. *“I’m uneducated, that’s all I know how to do (taking care of the elderly).”(P08)* Other than their wages, through communication with the participants, researchers learned that some family members provided additional funds to demonstrate their gratitude. This action generally created a sense of value and admiration among the participants. Therefore, compared with the past, many of the participants felt satisfied with their present life.

#### Being approved

The formal caregivers were considered the first point of contact, keeping a constant eye on their disabled elderly inpatients, being attentive and responsive to different needs, and offering 24/7 assistance with activities of daily living, such as toileting, transferring, and repositioning, as well as supportive emotional companionship. For some of these elderly patients, they felt more secure seeing their caregiver; if they did not, they felt uneasy. Formal caregivers are also needed by patients’ families. The family members, who were busy with their own jobs and daily lives, of the disabled elderly patients lacked the opportunity and the energy to provide sustained support for their own ill families, so they hired, and relied on the formal caregivers to take over their parents’ care. In addition, the formal caregivers often performed health-related tasks within the broader boundaries of their work content, such as monitoring and reporting the daily problems of their clients. Therefore, they navigated between the elderly patients in the hospital and were recognized and embraced by multiple stakeholders.

#### Emotion benefits

Caring for the elderly required patience, and the participants expressed love and respect for their clients and regarded them as their parents. When caring for the elderly, the participants who had lost their parents always thought of their own sick parents. More than half of the participants said that they had developed a strong bond with their clients after long periods of care. This deep feeling encouraged them to keep working.

### Theme 3:Responsibility Management

Nearly all the participants talked about responsibility because that was what they were paid for. After they got the job, they knew that they had to make promises to the disabled elderly’s family members. They felt that they had a duty to earn their wages and try to take care of the elderly, so they tried their best to explore the routines of their clients and the special skills needed to deal with a variety of challenges, which were the most important things to learn to succeed in their work. And the caregivers psychologically accept themselves by rationalizing the situation. Two sub-themes supported the theme of Responsibility Management: (1) Problem-Solving; (2) Psychological Acceptability.

### 3 − 1 Problem-solving

#### Regularity exploration

In order to take on the responsibility of caring for the elderly, the participants always tried their best to find ways of providing better care so that they could fit it into their work. Almost all participants expressed regularity exploration for older people, especially those with cognitive impairment, knowing that regularity is of the utmost importance. When they got the regularity of their clients, they assessed the patient regularly, and felt better about what they were doing. Many participants believed that it was difficult to find the right way to meet the needs of the clients until their temperament, physiology and habits were known.

#### Skills exploration

In the interview we learned that the formal caregivers had to deal with a variety of challenges, not only in meeting the habits of the clients, but also in exploring the special skills. There are two skills that are most commonly reported, one is how to care for very heavy patients who were much heavier than themselves. The other is how to handle the stool and urine of a disabled elderly person. In addition to discovering themselves, communicating with other formal caregivers is an important way of learning to explore skills.

### 3 − 2 Psychological acceptability

#### Rationalization

Some participants expressed that other pressures came from sexuality. They were shy about cleaning the bodies of patients of the opposite sex, especially the private parts. Instead of expressing hostility, the participants showed more compassion and empathy as the person in question was a patient. While some other pressures came from the abnormal behavior of the patients, such as hitting, shouting, and spitting, especially those with cognitive disorders. Such elderly patients were always treated both as ill and as kids. Participants expressed the truth that comforting them with warm words, praising and rewarding them, and letting them do what they wanted to do rather than controlling them, could make them calmer and more cooperative. To some especial clients who were unable to express themselves, participants expressed more sympathy and care. In terms of the elderly rolling over, formal caregivers helped their clients to roll over frequently and to keep their sheets and bedding clean and comfortable, even at night. They would put themselves in the patients’ shoes, and gave timely help to the elderly.

#### Religion

While, for caregivers with religious beliefs, such a sense of responsibility is even stronger. They always presented positive aspects and showed a willingness to keep trying and were happy to help others. In this study, two of the participants were religious, one a Christian and the other a Buddhist. Both thought it was normal to care for the elderly and expressed the will to make every effort to take care of the elderly.

#### Theme 4: Fear

The participants who spent their time in the hospital with their clients did not only see elderly people suffering from disease, but they also experienced mixed medical conditions such as costly medical expenses, rescue, death, busy children, and lonely patients. The biggest adaptive change is an increase in worrying about one’s own future, being disabled, ill and lonely, but no caregiver.

#### Fear of becoming a disabled elderly person

Formal caregivers, as frontline bystanders, can’t help but think, “What will I do when I grow old?” as they watch the helplessness and hopelessness of the sick elderly. This fear becomes even more obvious during their daily interaction with the elderly. Transitional anxiety also brings pessimistic thoughts. Some people even considered suicide if one day they were like their clients, believing that they would not have enough money to spend on treatments to make ends meet and would not want to be a burden on the family.

#### Fear of no followers

In addition, formal caregivers were also concerned about the possibility of incapacity leading to a loss of control over their own lives and having no one to care for them. They believed that taking care of a person with a disability was tiring, dirty, and a heavy burden, and not everyone was qualified to do this work, especially young people who were the only child in their family.

## Discussion

**Fig. 2:**
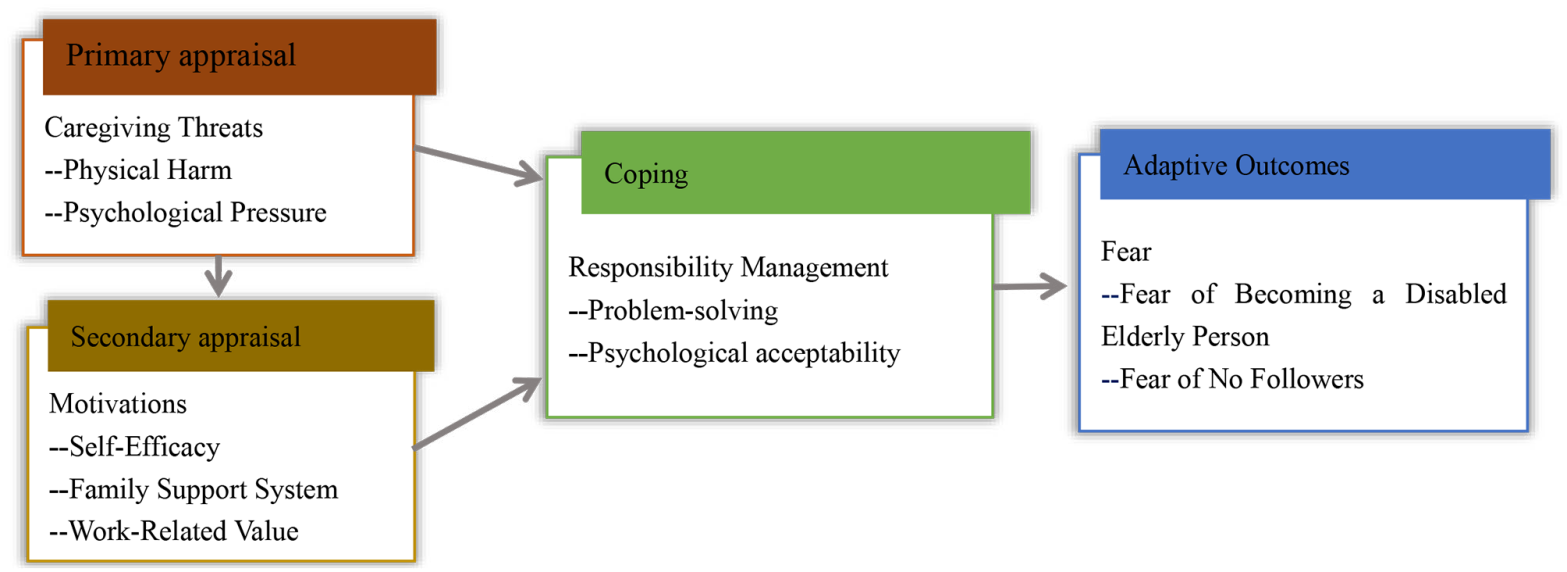
Based on the transactional model of stress and coping, formal caregivers initiate a process of primary appraisals (e.g. perceived threats) and secondary appraisals (e.g. motivations) in caring, which lead to coping efforts (e.g. responsibility management), while formal caregivers receive the adaptive outcomes (e.g. fear of becoming a disabled elderly person, and fear of no followers)

In the interviews, formal caregivers shared similar physical and mental treatment and difficulties. They have to perform some unwanted tasks, such as cleaning private parts and cleaning up urine, stool, or vomit, with no freedom and little dignity, sometimes, even having to endure criticism from clients. Just as previous literature has reported, formal caregivers experience significant physical and psychological strain [16, 18–21, 23]. More than that, in this study we found that in order to compensate for the heavy burden, they were still striving to maintain their internal and external motivations and responsibilities to actively explore a variety of coping strategies. Experiencing a range of conflicting emotions regarding their parents and customers, their financial stability being limited by their circumstances, and tedious working contents, these formal caregivers struggle with fear of their own future, as shown in the Fig. 2.

During the interviews, we found that the formal caregivers expressed their conflicts about feeling too exhausted to continue and having to keep their job. On the one hand, as the “sandwich generation” [47], with age limits, low levels of education, and work demands, they also bore the responsibility of supporting their young offspring and elderly parents. Pay satisfaction was seen as an intrinsic motivation to stay on the job, a finding in line with that in other studies [48], although some participants felt guilty about not caring for their own parents. While, on the other hand, many of the participants enjoyed being with the disabled elderly patients as if they were with their own family members, as was found in a previous study [49], and during the care process they attained emotional benefits [50]. Furthermore, they were recognized and respected for their work by clients’ families or medical staff, which also motivated them to continue their work despite the difficulties they face. Therefore, in this study, the participants expressed that although they suffered great pressure, they would hold on and keep going.

The study found that conscience and health were important elements of caregivers’ competence, and you can’t do without either. Almost all of them mentioned “conscience”, which is mainly reflected in the attitude towards the elderly, with responsibility, patience and love. Therefore, these caregivers tried to find various ways to cope with physical and psychological burdens actively, like rationalizing the disabled elderly patients’ abnormal behavior, exploring some tricks and regularity to cater for their clients, and finding benefits to relieve them. The study also found that if the formal caregiver was optimistic, had more optimal communication with stakeholders, felt value in their work, and had a good family support system, they had a higher level of professional identity, and a lower level of mental stress and strain. Otherwise, they showed low levels of professional identity, pessimism and deeper feelings of guilt for neglecting their own families. These findings are similar to those in previous studies [24, 51], formal caregivers’ personality traits and the attitudes of the people around them directly affect how they cope with the heavy burden. In addition, in the present study another interesting phenomenon was that for the participants who had good sleep habits (i.e., those who fell asleep the moment their head touched the pillow) tended to have more positive, proactive thinking in terms of problem-solving and emotional coping, despite waking up frequently during the night. In contrast, others with poor sleep habits were more passive, pessimistic, and impatient. Poor sleep quality has a direct positive impact on depressive symptoms [52]. But no matter what, when formal caregivers navigated between patients in the hospital and saw the pain caused by diseases, the huge medical expenses, the helplessness and loneliness of the disabled elderly, and even death. They were under a great deal of stress and fear. They feared getting sick, becoming disabled, not being cared for and burdening their families.

## Study limitations

This study also has some limitations. The study included only individuals from a tertiary general hospital in Hangzhou, China, within a specific geographical region. In terms of interviewees and sampling, we selected participants by purposive sampling with maximizing variation. However, the majority of participants were female, reflecting the limited number of male formal caregivers in China. Despite reaching saturation, our sample size remains small. It could be imperfect in terms of its representation. Moreover, there was a potential for recall bias as participants were asked to remember and describe their caregiving experiences. While in terms of gathering and analyzing data, the researchers may have infused their subjective assessments and hypotheses.

## Conclusion

This study is important because it complements previous research by showing that the experiences of formal caregivers of older people with physical disabilities in hospital are two-sided. Even under immense work pressure, formal caregivers of elderly inpatients with physical disabilities exhibit determination and a range of coping mechanisms to succeed in their responsibilities keenly. Furthermore, they also experienced emotional benefits from their work, not just the income gained. The findings indicate the importance of offering timely support and feedback to individuals in this profession, as it can enhance their ability to handle stress and alleviate anxiety. Stable formal caregivers are essential for the well-being of people with physical disabilities. In the future, on the one hand, further quantitative studies with large sample sizes are needed to confirm and extend the feelings and experiences of formal caregivers revealed in this study. On the other hand, more effective improvement strategies are needed to build a sustainable and high quality care system.

## Data Availability

The data sets that were used and/or analyzed in the study are available on request from the corresponding author.
